# The influence of thyroid diseases, diabetes mellitus, primary hyperparathyroidism, vitamin B12 deficiency and other comorbid autoimmune diseases on treatment outcome in patients with rheumatoid arthritis: An exploratory cohort study

**DOI:** 10.1097/MD.0000000000010865

**Published:** 2018-05-25

**Authors:** Amir Emamifar, Inger Marie Jensen Hansen

**Affiliations:** a Department of Medicine, Odense University Hospital, Svendborg Hospital, Svendborg; b Faculty of Health Sciences, University of Southern Denmark, Odense; c Section of Rheumatology, Department of Medicine, Odense University Hospital, Svendborg Hospital, Svendborg; d Danbio, Copenhagen, Denmark.

**Keywords:** autoimmune diseases, comorbidity, disease activity score in 28 joints-C-reactive protein, rheumatoid arthritis

## Abstract

To investigate the impact of comorbid diseases on rheumatoid arthritis (RA) outcome.

All patients diagnosed with RA since 2006, who were registered in our local Danbio registry, were included in this cohort study. Patients’ demographics, serology results, and Disease Activity Score in 28 joints-C-reactive protein (DAS28-CRP) at the time of diagnosis and after 4 months of treatment initiation were collected. Patients’ electronic hospital records were evaluated for a positive history of thyroid diseases, diabetes mellitus, primary hyperparathyroidism, vitamin B12 deficiency, and the presence of other diagnosed autoimmune diseases.

1035 RA patients were included. The observed prevalence of thyroid diseases was 11.8%, DM 10.4%, primary hyperparathyroidism 2.8%, vitamin B12 deficiency 5.8%, and other diagnosed autoimmune diseases 1.6%. There were significant associations between presence of thyroid diseases and female gender (*P < *.001); DM and greater age (*P < *.001); primary hyperparathyroidism and longer disease duration (*P = *.002); other diagnosed autoimmune diseases and antinuclear antibody positivity (*P < *.001). RA patients with thyroid diseases (*P = *.001) and other comorbid autoimmune diseases (*P < *.001) had significantly poorer initial response to the RA treatment compared to patients with isolated RA.

Univariate analyses revealed that age, the presence of thyroid diseases, the presence of other diagnosed autoimmune diseases and DAS28-CRP at the time of diagnosis were significantly associated with ΔDAS28-CRP. Additionally, multivariate analysis demonstrated that ΔDAS28-CRP deterioration was significantly correlated to the presence of thyroid diseases (unstandardized regression coefficient (standard error); −0.188 (0.088), *P = *.030) and the presence of other diagnosed autoimmune diseases (−0.537 (0.208), *P = *.010).

RA patients are at increased risk of specific comorbidities with possible impact on the treatment outcome. To improve this situation, periodic assessment of comorbidities should be considered.

## 1 Introduction

Rheumatoid arthritis (RA) is a chronic inflammatory disease, with a prevalence of 0.5% to 1% in the general population that predominantly affects joints. However, patients with RA may present with extra articular presentations.^[1–2]^ Additionally, there are various comorbidities that can complicate the course of RA disease. The most common comorbidities among patients with RA are cardiovascular events, infections, pulmonary diseases, different types of cancers, depression, etc.^[3]^ Besides, some of these comorbidities are less frequently discussed in the literature, for example, hearing loss.^[4]^ RA comorbidities are associated with loss of function, higher rate of hospitalization, increased mortality rate as well as socioeconomic burden on the patients and society.^[3,5–6]^

The association between comorbid diseases and RA is a complex dilemma. Comorbid diseases may present prior, later or at the same time. They might be a consequence of RA treatment, for example, corticosteroids or a predisposing factor can lead both to RA and comorbid diseases, for example, smoking.^[3,5]^ Furthermore, RA comorbidities may present due to a shared autoimmune pathology, described as polyautoimmunity or multiple autoimmune syndrome.^[7]^ RA comorbidities are often underdiagnosed and undertreated.^[8,9]^

Given the importance of comorbid diseases in RA, it is important to diagnose and subsequently treat such conditions in RA patients, which is recommended by the European League Against Rheumatism (EULAR).^[10]^ In addition, thorough investigation of comorbid diseases in RA can also aim to understand the common pathological association.

Disease Activity Score in 28 joints-C-reactive protein (DAS28-CRP) is a scoring system that is commonly used to evaluate treatment response as well as monitoring disease activity in clinical practice. It is derived from 2 subjective parameters, that is, tender joints (TJ) count and patient global assessment and 2 objective parameters, that is, swollen joints (SJ) count and laboratory value of CRP.^[11]^ DAS28-CRP is a valuable tool to optimize outcome in RA patients, by means of measuring disease activity and thereafter adjusting treatment, that is, “treat-to-target.”^[11]^

The primary objective of this study was to reveal the prevalence of important comorbidities i.e thyroid disease, diabetes mellitus (DM), primary hyperparathyroidism, and vitamin B12 deficiency as well as other comorbid autoimmune diseases in our RA patients. Furthermore, we investigated the possible associations between clinical characteristic of RA and these comorbidities. At last, the effect of these comorbidities on initial treatment response was evaluated with the aim of DAS28-CRP, since the initial treatment response is an independent prognostic factor.^[12–14]^

The relationship between RA and thyroid diseases has been discussed in previous studies, in which indicates higher prevalence of thyroid diseases in RA patients compared to the general population (about 2–3 times).^[15–17]^ The most accepted pathology is autoimmunity where human leukocyte antigen (HLA) gene complex plays a significant role.^[18]^

There are inconsistencies regarding the prevalence of DM in RA, however the previous research support the increased prevalence of DM or insulin resistance in RA in many instances, caused by immune system activation and/or RA treatment.^[19,20]^ Tumor necrosis factor alpha (TNF-a) is a mediator of insulin resistance and has a major role in the pathogenesis of RA.^[21,22]^ Additionally, TNF-a inhibitors reduces the risk of developing DM in RA patients, in which emphasizes the involvement of TNF-a in the common pathogenesis between RA and DM.^[23]^

A connection between vitamin b12 deficiency and RA has been suggested before, probably due to deficient nutrition and eventual malabsorption secondary to autoimmune mechanisms.^[24–26]^ Vitamin B12 may result in hyperhomocysteinemia which is an independent risk factor for cardiovascular disease.^[27,28]^ The prevalence of vitamin B12 deficiency in patients with RA is variable. This was reported equal to 4% by Pettersson et al, 24.9% by Segal et al, and 30% by Vreugdenhil et al.^[24,29,30]^

Primary hyperparathyroidism is a metabolic disorder of one or more of the parathyroid glands with a prevalence of 1 to 7 per 1000 adults.^[31]^ The presence of primary hyperparathyroidism in RA patients may aggravate the effect of RA on bones and joints by means of interaction with cytokines and inflammatory markers involved in RA.^[32]^

## 2 Materials and methods

### 2.1 Danish danbio registry

The Danish Danbio registry was firstly established in 2000. It provides nationwide data on the disease course of patients with inflammatory rheumatic disease including RA via unique personal identification code. Danbio has been approved by The Danish Data Registry (j. nr. 2007-58-0014 and j. nr. 2007-58-0006), and National Board of Health (j. nr. 7-201-03-12/1) and thereafter, since 2006, it became mandatory to report to the registry why all newly diagnosed patients as well as patients referred from other departments have been registered in Danbio. Data are collected from patients and health personnel (nurses and physicians) and are basically divided to baseline variables (e.g., demographic data, diagnosis, diseases duration) and Longitudinal/follow up data (e.g., treatment, functional status, and disease activity scores).^[33]^ Each section of rheumatology has access to its own patients. At our section of rheumatology, all patients with diagnosis of RA are registered in Danbio at every consultation.

### 2.2 Study design and settings

This is an observational cohort study. The whole parts of the study were performed at the section of rheumatology, Svendborg Hospital in December 2016. The study was approved by Danish Data Protection Agency (file no. 14/50243) and Danish Patient Safety Authority (file no. 3-3013-1542/1/).

### 2.3 Participants

All patients with diagnosis of RA registered in Danbio since 2006, were considered to enter into the study. The diagnosis of RA was established according to the 1987 American College of Rheumatology (ACR) criteria for RA (old criteria) and, since 2010, based on the 2010 ACR/EULAR criteria for RA (new criteria).^[34,35]^ Inclusion criteria were as follows: Patients who were registered at the rheumatology section of Svendborg hospital, age ≥ 18 years old. Patients who passed away or were referred to the other departments were also included in the study. Patients with juvenile RA were excluded from the study.

### 2.4 Initial rheumatoid arthritis treatment

At our section of rheumatology, patients with newly established diagnosis of RA are initially treated with methotrexate, which may be increased to 25 mg per week, depends on DAS28-CRP as an index of disease activity. Furthermore, treatment can be supplemented by hydroxychloroquine and sulfasalazine as well as prednisolone (given Intramuscular, intra-articular or orally) in case of persistent inflammation. The treatment goal is to achieve remission, that is, DAS28-CRP <2.6 (or low disease activity, i.e., DAS28-CRP≤3.2) as quickly as possible.

### 2.5 Data collection

Patients’ demographic data (age, sex, year of diagnosis), disease duration, serology test results including immunoglobulin M rheumatoid factor (IgM-RF), anticyclic citrullinated peptide antibody (anti-ccp), and antinuclear antibody (ANA) were extracted. DAS28-CRP at the time of diagnosis and after 4 months (±1–2 months) of treatment initiation was also collected. The electronic hospital records of the patients for the last 10 years (since 2006) were evaluated, to the extent data were available, for a positive history of thyroid diseases, DM, primary hyperparathyroidism, vitamin B12 deficiency as well as the presence of other diagnosed autoimmune diseases which is described with details below:

#### 2.5.1 Thyroid diseases

To detect thyroid diseases in this study, we searched our patients’ electronic hospital records for any positive history of thyroid diseases. Furthermore, results of thyroid laboratory tests (triiodothyronine [T3], thyroxine [T4], thyroid stimulating hormone [TSH]), as well as patients’ medication list were reviewed to find any abnormal lab results or use of thyroid medications to find comorbid thyroid diseases. Diagnosis of thyroid diseases were made at the section of endocrinology based on routine follow up and updated guidelines. Patients with subclinical thyroid disease (increased or decreased TSH, normal T3/T4, no clinical symptoms) were not considered to estimate the prevalence of thyroid diseases, nor used to perform other statistical analysis. The individual medical records from family physicians were not examined in this study, since we did not have access to such records.

#### 2.5.2 Diabetes mellitus

Considering the possible relationship between RA and DM, the electronic hospital records of the patients were reviewed, in a similar way, for a positive history of DM as well as prescribed antidiabetic medications and abnormal lab tests (increased fasting blood sugar [FBS] and hemoglobin A1C [HbA1C]) to identify whether the patients had been diagnosed with DM as well. Types of DM were extracted from Fyns Diabetes Database.

#### 2.5.3 Primary hyperparathyroidism

Patients’ electronic hospital records including laboratory results (parathyroid hormone [PTH] and calcium levels) were reviewed to reveal if they had been diagnosed with primary hyperparathyroidism. According to the Danish endocrinology society, primary hyperparathyroidism can be diagnosed as follows:

A plasma calcium concentration above the upper reference range, where a plasma PTH concentration is measured in the upper third or above the upper limit of the reference range. With the 2nd generation of PTH assays, which is most commonly used, a plasma PTH concentration >5 pmol /L will be considered to be a disproportionately high PTH concentration in a patient with hypercalcemia. Hyperparathyroid hypercalcemia state should be observed with at least 2 measurements and, besides, other causes of hyperparathyroid hypercalcemia should be excluded (e.g., familial hypocalciuric hypercalcemia, thiazide diuretics or lithium therapy as well as tertiary hyperparathyroidism).^[36]^

#### 2.5.4 Vitamin B12 deficiency

Diagnosis of vitamin B12 deficiency was established with respect to the serum level of vitamin B12 and methylmalonic acid (MMA). The vitamin B12 deficiency was considered as vitamin B12 < 148 pmol/L (<200 pgr/mL) as also suggested by previous studies. In case of vitamin B12 between 148 to 258 pmol/L (201–350 pg/mL) MMA was used to confirm diagnosis. In addition to the laboratory results, patients’ electronic hospital records and medication list were also reviewed to find patients with comorbid vitamin B12 deficiency.

#### 2.5.5 Other diagnosed autoimmune comorbidities

During data extraction, any other diagnosed autoimmune diseases were found in patients’ electronic hospital records were collected. Thereafter, patients autoantibodies profile were also assessed to explore any comorbid autoimmune diseases. Patients with only positive autoantibodies profile and without definite diagnosis were not considered to perform statistical analysis.

### 2.6 Variables

Demographic data were extracted from Danbio. The results of IgM-RF (normal range: < 15 IU/mL), anti-ccp (normal range: < 20 EU/mL), and ANA (normal range: < 1/0 IU) were collected and analyzed both quantitative and qualitative (positive/negative). DAS28-CRP and ΔDAS28-CRP were calculated as follows:

DAS28-CRP = 0.56^∗^√(Tender Joint) + 0.28^∗^√(Swollen Joint) + 0.36^∗^ln (CRP+1) + 0.014^∗^Global Visual Analog Scale + 0.96.

ΔDAS28-CRP = DAS28_1_-CRP (at the time of diagnosis) – DAS28_4_-CRP (after 4 months of treatment initiation ± 1–2 months) representing initial treatment response. The lower reporting limit of CRP was considered as < 10 mg/L.^[37]^

The local laboratory reference values were as follows: TSH (0.3–4 mIU/L), T3 (1.3–2.2 nmol/L), T4 (60–130 nmol/L), FBS (<126 mg/dL), HbA1C (<6.5%), PTH (1.1–6.9 pmol/L), calcium (1.19–1.29 mmol/L), vitamin B12 (130–700 pmol/L), and MMA (0.08–0.28 μmol/L).

### 2.7 Statistical analysis

All statistical analyses were performed using IBM SPSS Statistics version 24.0. Continuous data were presented as mean ± standard deviation (±SD), categorical data as frequencies and respective percentages. Comparisons of baseline demographics and RA related characteristics, between groups with and without specific comorbidity were made with Student's *t*-test. When comparing 2 binary variables the Chi-square was performed. The *P* value was considered as significant if *P* < .05. In case of missing data, we used pairwise deletion to keeps as many cases as possible for each analysis.

Univariate and multivariate analysis were performed to delineate the relationship between the dependent variable, that is, ΔDAS28-CRP and independent variables including: gender, age, IgM-RF, anti-ccp, ANA, disease duration, presence of thyroid diseases, presence of primary hyperparathyroidism, presence of DM, presence of vitamin B12 deficiency, presence of other diagnosed autoimmune diseases, TSH level, and DAS28_1_-CRP. Hereafter, variables were removed from the multiple regression models in a backward fashion, based on the results of multivariate analysis and supposed clinical relevance. The risk for DAS28-CRP deterioration after 4 months of treatment (±1–2 months), that is, ΔDAS28-CRP is expressed as unstandardized regression coefficient, standard error and a level of significance (p value).

## 3 Results

Around 1035 patients with diagnosis of RA were included in the study. A total of 23 patients with juvenile RA/unspecified juvenile arthritis were excluded from the study. Patients’ demographic and clinical characteristics are summarized in Table 1.

**Table 1:**
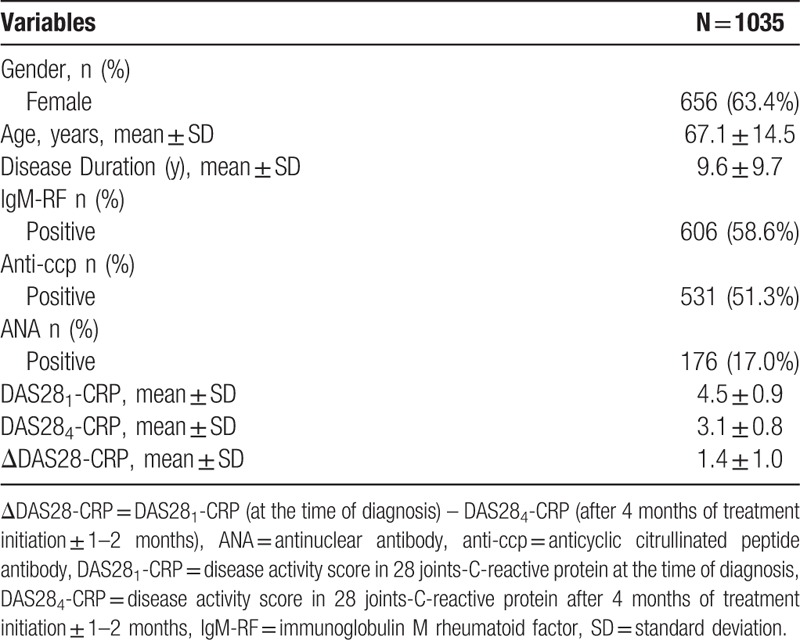
Patients’ demographic and clinical characteristics.

### 3.1 Prevalence of comorbidities

The overall prevalence of different comorbidities in our RA population is listed in Table 2.

**Table 2:**
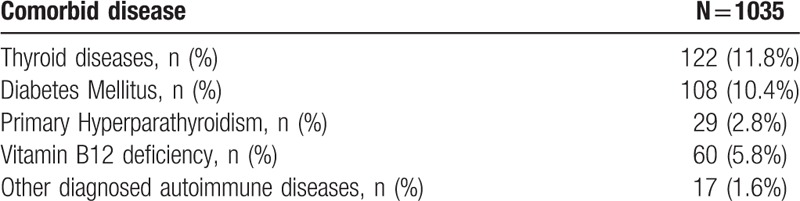
Prevalence of different comorbidities in RA patients.

Thyroid diseases were found in 122 (11.8%). Hypothyroidism was the most common thyroid dysfunction (74/122 (60.6%)) in our RA patients. Of 108 RA patients with DM, 94 (87%) and 14 (13%) patients were diagnosed with type II and type I, respectively. Sjogren's syndrome (10/17 [58.8%]) was most prevalent comorbid autoimmune diseases followed by inflammatory bowl disease (4/17 (23.5%)), systemic sclerosis (1/17 [5.9%]), celiac disease (1/17 [5.9%]), primary biliary cirrhosis (1/17 [5.9%]). In addition to the 17 patients with other diagnosed autoimmune diseases, 28 patients had positive autoantibodies profile; however, the diagnosis of comorbid diseases were not made at the time of the study and patients were under further work up.

### 3.2 Comparison of baseline demographics and RA related characteristics in patients presented with/without each specific comorbidity

Comparison of baseline demographics and RA related characteristics in patients presented with/without thyroid disease, DM, primary hyperparathyroidism, vitamin B12 deficiency and other diagnosed autoimmune diseases is summarized in Table 3.

**Table 3:**
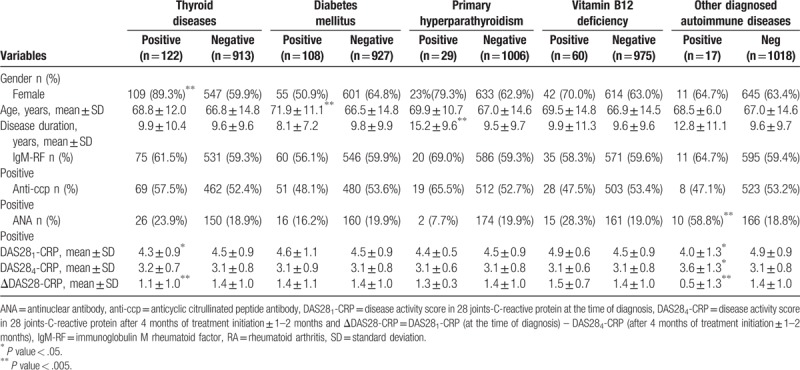
Comparison of baseline demographics and RA related characteristics in RA patients presented with/without thyroid disease, diabetes mellitus, primary hyperparathyroidism, vitamin B12 deficiency and other diagnosed autoimmune diseases.

There were significant associations between presence of thyroid diseases and female gender (*P* value < .001); DM and greater age (*P* value < .001); primary hyperparathyroidism and longer disease duration (*P* value = .002) as well as other diagnosed autoimmune diseases and ANA positivity (*P* value <.001).

RA patients with thyroid diseases (*P* value = .001) and other comorbid autoimmune diseases (*P* value < .001) had significantly poorer initial response to the RA treatment compared to patients with isolated RA.

Univariate analyses revealed that age, the presence of thyroid diseases, the presence of other diagnosed autoimmune diseases, and DAS28_1_-CRP were significantly associated with ΔDAS28-CRP (Table 4).

**Table 4:**
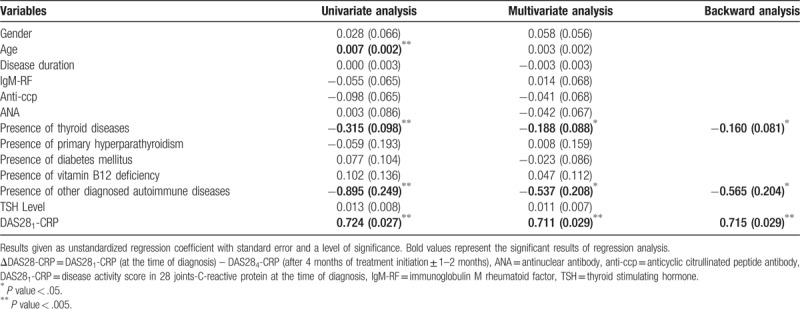
Univariate and multivariate analysis of risk factors for ΔDAS28-CRP.

Additionally, multivariate analysis demonstrated that ΔDAS28-CRP deterioration was significantly correlated to the presence of thyroid diseases (unstandardized regression coefficient (standard error); −0.188 (0.088), *P* value = .030) and presence of other diagnosed autoimmune diseases (−0.537 [0.208], *P* value = .010). There was also a positive correlation between ΔDAS28-CRP and DAS28_1_-CRP (0.711 [0.029], *P* value < .001).

In the backward-stepwise regression analysis, the presence of thyroid diseases, presence of other diagnosed autoimmune diseases and DAS28_1_-CRP remained in the model.

## 4 Discussion

In this cohort study, the observed prevalence of thyroid diseases in our center was 11.8%, DM 10.4%, primary hyperparathyroidism 2.8%, vitamin B12 deficiency 5.8%, and other diagnosed autoimmune diseases 1.6%. We also found significant associations between presence of thyroid diseases and female gender; DM and greater age; primary hyperparathyroidism and longer disease duration as well as other diagnosed autoimmune diseases and ANA positivity. Furthermore, the results of our study suggested that the initial RA treatment response was significantly poorer among RA patients with thyroid diseases and other diagnosed autoimmune diseases compared to the patients with isolated RA.

In the present study, we explored the ΔDAS28-CRP index representing initial response to RA treatment after 4 months (±1–2 months) of treatment. Indeed, the concept of ΔDAS28-CRP implemented on the importance of first few months after treatment initiation has been suggested previously.^[12]^ It has been accepted that patients with RA should be diagnosed and treated promptly to prevent further joint destruction. The first few months succeeding treatment initiation are pivotal for RA long-term outcome.^[12]^ A better long-term outcome can be gained by means of a lower disease activity at 6 months of treatment. Furthermore, a clinical remission achieved within 3 to 6 months of RA treatment, regardless of treatment regime, halts the progression of joint damage.^[13,14]^ Longer follow-up in future studies is recommended which may add more information to our findings.

We previously demonstrated that the prevalence of thyroid diseases and DM were higher among a group of RA patients who were diagnosed according to the new 2010 ACR/EULAR criteria for RA.^[15,38]^. Results of the present study are in line with our previous findings. Primary hyperparathyroidism in our RA population was found 3 times more common than in the general population, considering the increasing prevalence rate in the elderly (up to 1 per 100 in the elderly).^[39,40]^ The link between primary hyperparathyroidism and RA is poorly described in the literature and is limited to few case reports and old data.^[41–43]^ This might represent the coincidence of 2 common diseases, as suggested by Crisp et al, or due to the potential effect of enteric hormones on the calcium metabolism.^[44]^

As expected, there was a tendency to develop vitamin B12 deficiency in our RA patients (5.8% of the patients). This is of importance due to the fact that hyperhomocysteinemia is an independent risk factor for cardiovascular disease, considering the fact that RA patient are seemingly at high risk of cardiovascular morbidity.^[45]^

Among RA patients with other autoimmune diseases, Sjogren's syndrome was the most prevalent disease. Sjogren's syndrome is a common condition in RA.^[46]^ The prevalence of Sjogren's syndrome was lower than that of reported prevalence in the literature (1% vs 4–31%). This might be due to the fact that we only considered patients whom the diagnosis of Sjogren's syndrome was made prior to the conduct of the study. Some of the patients were under further work up at the time of data extraction.

Polyautoimmunity has been defined as a presence of more than one well defined autoimmune disease in an individual patient. This should be differentiated from overlapping syndrome that is a partial presence of clinical signs/symptoms of various autoimmune diseases. Multiple autoimmune syndrome is used, when 3 or more autoimmune diseases exist at the same time. Polyautoimmunity is common in RA and influenced by clinical and immunological features.^[47]^ However the authors presume that the shared autoimmunity is not the only cause of increased prevalence of comorbidities seen in this study. Genetics, gender, environmental factors may play roles in the presence of one or more comorbidities in RA patients.

Of great interest to us, the presence of thyroid diseases and other autoimmune diseases in our RA population were correlated with worsening of the DAS28-CRP after 4 months (±1–2 months) of treatment initiation. This confirmed our previous findings which were indicative of worse initial treatment outcome in patients with newly diagnosed RA patients with thyroid diseases where diagnosis of RA was made according to the 2010 ACR/EULAR criteria for RA.^[15]^ In terms of other diagnosed autoimmune diseases, for example, Sjogren's syndrome, earlier studies were suggestive of poor RA outcome with more complications and systemic involvement in RA patients with diagnosed Sjogren's syndrome.^[46]^

The strengths of our study were the large sample size and broad inclusion criteria which minimize the selection bias. Besides, we explored ΔDAS28-CRP during the first 4 (±1–2 months) months after start of treatment, since the initial treatment response is a prognostic factor. Though the prior mentioned strengths, there were some limitations. The comorbidities reported in the study were selected by the authors and did not include all types of comorbidities. Other types of comorbidities may or may not affect the treatment outcome as well as the prevalence of the mentioned comorbidities in our study. We did not include RA patients from general practices as well as rheumatologists in private practice, since these patients, usually with mild disease, were not being referred to the hospital. Furthermore, comorbid diseases diagnosed longer than 10 years ago were not identified in this study which may underestimate the prevalence of comorbid diseases. With respect to the prevalence of different comorbidities in our study, the prevalence of some of these comorbidities, on the other hand, might be overestimated due to diagnostic or reporting bias. Probably, patients with RA are more frequently offered to screen for recognized comorbidities. Besides, they may more commonly be diagnosed with comorbidities that a known association has been found between these comorbidities and RA. The authors suggest performing a prospective study with a comparator group without RA to delineate the effect of mentioned comorbidities in this study, specifically, thyroid diseases and other autoimmune diseases, to confirm our results. The results of this study have a high degree of generalizability due to broad inclusion criteria.

In conclusion, we provide evidence that patients with RA are at increased risk of specific comorbidities. These comorbidities may affect the outcome of patients with RA. To improve this situation, periodic assessment of comorbidities should be kept in mind. We recommend to measure TSH and HbA1C on a yearly basis in all RA patients to diagnose concurrent thyroid diseases or DM. Furthermore, we recommend assessment of autoantibodies directed against Ro/SSA and La/SSB autoantigens as soon as patients present with sicca symptoms (dry eyes and dry mouth). If calcium level is continuously elevated, measurement of PTH should be taken into account. In case of megaloblastic anemia, vitamin B12 deficiency should be suspected and laboratory measurement of vitamin B12 level and methylmalonic acid should be requested.

## Acknowledgment

We thank Dr Rikke Assmussen Andreason, Dr Rasmus Hviid Larsen, and Mrs Maryam Mousavi for their contribution to data collection. We also thank Danbio.

## Author contributions

**Conceptualization:** Amir Emamifar, Inger Marie Jensen Hansen.

**Data curation:** Amir Emamifar.

**Formal analysis:** Amir Emamifar.

**Methodology:** Amir Emamifar, Inger Marie Jensen Hansen.

**Supervision:** Inger Marie Jensen Hansen.

**Validation:** Amir Emamifar, Inger Marie Jensen Hansen.

**Writing – original draft:** Amir Emamifar, Inger Marie Jensen Hansen.

**Writing – review & editing:** Amir Emamifar, Inger Marie Jensen Hansen.
